# Laser-Induced Transferred Antibacterial Nanoparticles for Mixed-Species Bacteria Biofilm Inactivation

**DOI:** 10.3390/ma16124309

**Published:** 2023-06-11

**Authors:** Alena Nastulyavichus, Eteri Tolordava, Sergey Kudryashov, Roman Khmelnitskii, Andrey Ionin

**Affiliations:** 1 P. N. Lebedev Physics Institute of Russian Academy of Sciences, 119991 Moscow, Russiaioninaa@lebedev.ru (A.I.); 2 School of Natural Sciences and Mathematics, Ural Federal University, 620000 Ekaterinburg, Russia

**Keywords:** antibacterial nanoparticles, mixed-species bacterial biofilms, LIFT, bactericidal effect

## Abstract

In the present study, copper and silver nanoparticles with a concentration of 20 µg/cm^2^ were synthesized using the method of laser-induced forward transfer (LIFT). The antibacterial activity of the nanoparticles was tested against bacterial biofilms that are common in nature, formed by several types of microorganisms (mixed-species bacteria biofilms): *Staphylococcus aureus, Escherichia coli*, and *Pseudomonas aeruginosa.* The Cu nanoparticles showed complete inhibition of the bacteria biofilms used. In the course of the work, a high level of antibacterial activity was demonstrated by nanoparticles. This activity manifested in the complete suppression of the daily biofilm, with the number of bacteria decreasing by 5–8 orders of magnitude from the initial concentration. To confirm antibacterial activity, and determine reductions in cell viability, the Live/Dead Bacterial Viability Kit was used. FTIR spectroscopy revealed that after Cu NP treatment, there was in a slight shift in the region, which corresponded to fatty acids, indicating a decrease in the relative motional freedom of molecules.

## 1. Introduction

Bacterial resistance to antibiotics and various disinfectants is currently a serious global problem. Most bacteria have the ability to form biofilms, which are a conglomerate of microorganisms attached to any surface, and united by an extracellular matrix (which includes polysaccharides, proteins, and DNA) [1,2]. Bacterial biofilms can form anywhere, including on medical instruments such as catheters and implants [1,3]. Biofilms are responsible for the majority of chronic infections, which are mainly associated with the use of medical implantable equipment [4,5,6], and do not respond to antibiotic treatment [6].

Among the best-known clinical microorganisms that form biofilms are *Staphylococcus* spp., *Pseudomonas aeruginosa* [7]. These bacteria are opportunistic pathogens that are most often isolated from chronic wounds. Despite early work suggesting that one bacterial species would outperform another at the stage of biofilm formation, recent studies have shown that two bacteria species can coexist [8].

One of the tasks involved in the study of mixed biofilms is the study of the growth of a mixed biofilm, and how bacteria interact with each other. The results of studies [9] point to clear interactions between *Staphylococcus aureus (S. Aureus.)* and *Pseudomonas aeruginosa (P. Aeruginosa.)*, which are both competitive, and mutually beneficial for each organism, in terms of pathogenicity and colonization. In the context of a wider range of studies describing both in vitro and in vivo interactions between *S. Aureus*. and *P. Aeruginosa*, it has been suggested that these bacteria interact at an early stage for the best chance of colonization; however, they are then separated.

Most antibacterial studies are carried out on single biofilms [10,11,12,13,14]. However, in reality, mixed biofilms are of the greatest interest. Let us consider some studies on methods of dealing with mixed biofilms. According to the authors of [15,16,17], the most effective method for combating mixed biofilms is cold atmospheric plasma. In [18], it was shown that combination therapy, including antifungal and antibacterial agents, can be a very useful strategy to achieve the prevention and control of mixed biofilms. Zhen-quan Yang et al. suggested that the combination of phages with other green technologies could provide practical alternatives to traditional methods of dealing with mixed biofilms [19]. Milho et al. [20] reported on the effectiveness of phages and interspecies interactions against biofilms formed by two species, *E. coli* and *S. enteritidis*. Another method is the use of ultraviolet radiation [21]. One work [22] aimed to explore the anti-biofilm efficacy of sonodynamic therapy using nano-emodin against mixed-species bacterial biofilms containing *Staphylococcus aureus, Pseudomonas aeruginosa*, and *Acinetobacter baumannii*. Bacterial biofilms can be destroyed using polyhexamethylene biguanide, via a disrupting of the membrane [23].

Metal nanoparticles are a powerful bactericidal agent with a wide spectrum of action, determined by a number of possible mechanisms of action on living cells: the generation of reactive oxygen species, the release of biotoxic metal ions, the blocking of metabolic channels, and electrostatic and nanomechanical effects [24]. Unlike antibiotics, each of which affects a specific target in a microbial cell; such as the cell wall, the cytoplasmic membrane, DNA replication, transcription, or translation of proteins; generated reactive oxygen species cause oxidative damage to components of both a lipid and protein nature, as well as nucleic acids. It is believed that the multiple oxidative nature of this damage to cellular components prevents the development of resistance; therefore, this method is considered a promising way to combat pathogens that are resistant to the action of traditional drugs.

The paper [25] showed that copper nanoparticles obtained by laser ablation in liquid have high antibacterial efficiency against *Staphylococcus aureus*. Another work [26] demonstrated the antibacterial properties of copper nanoparticles on *Escherichia coli.* The research demonstrated that Cu NPs inhibited bacterial growth and that Cu NP-coated surfaces decreased the microbial count and the microbial biofilm formation. The research [27] showed that silver nanoparticles, synthesized by chemical reduction method, have strong antibacterial and antibiofilm activity. The highest inhibitory and destructive effect on biofilms was exhibited by Ag NPs prepared using an extract from L. indica [28]. Silver nanoparticles showed good efficiency in relation to the binary biofilm of *S. aureus, C. albica* [29]. Additionally, ZnO nanoparticles have proven effective against the biofilm of *S. aureus/E. coli* [30]. In [31], hybrid silver/gold nanoparticles were developed as antimicrobial agents to combat the formation of biofilms. The studies were conducted on a mixed *Staphylococcus aureus–Pseudomonas aeruginosa* biofilm. Intracellular infection was reduced by 70–90% in fibroblast and monocyte cell lines.

In this work, we applied the LIFT (laser-induced forward transfer) method of biofilm inactivation of bacteria, which has proven itself previously on single biofilms [10,11]. Microbiological studies have shown the complete death of daily mixed-species biofilms formed by two bacteria: gram-positive *Staphylococcus aureus* and gram-negative *Pseudomonas aeruginosa* and *Escherichia coli*. The Live/Dead method was used to confirm the results.

## 2. Materials and Methods

To conduct this research, we used biofilms of museum strains of gram-positive *Staphylococcus aureus 15* and gram-negative bacteria *Pseudomonas Aeruginosa 32* and *Escherichia coli 697* obtained from the working collection of N. F. Gamaleya Federal Research Center of Epidemiology and Microbiology. Based on the main principles of the O’Toole method, bacterial biofilms were grown on glass substrates (Glass CΠ-7105, chemical composition: SiO_2_ CaO MgO Al_2_O_3_ Na_2_O).

To do this, a fresh (daily) broth culture was diluted in a ratio of 1:100 in the nutrient medium of Luria–Bertani (LB) (Miller, AppliChem, Germany), and poured into test tubes with glass substrates. To obtain mixed biofilms at the initial stage of biofilm formation, we mixed cultures 1:1, and the resulting mixture of cultures was diluted 1:100 in a nutrient medium. Then, the biofilms were grown at 37 °C for 24 h.

**Figure 1:**
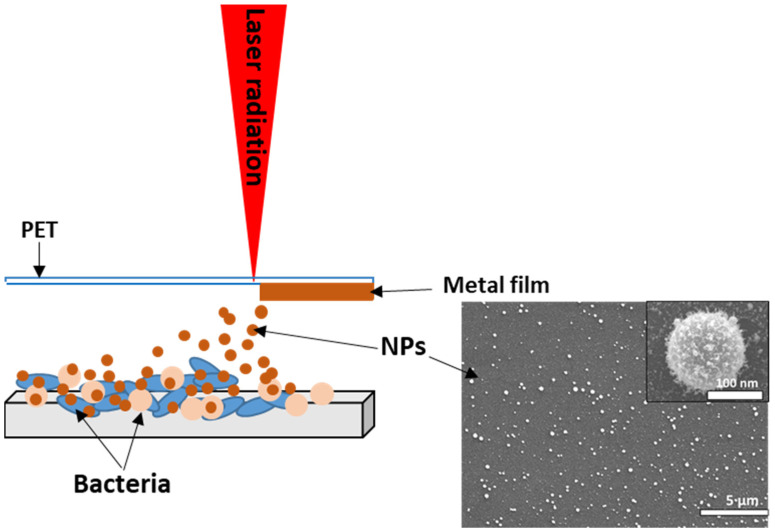
Scheme of the experiment. Inset: SEM visualization of Cu NPs.

The bacterial biofilms were treated with silver (99.99%) and copper (99.99%) nanoparticles obtained using the laser-induced forward transfer of a thin metal layer from polyethylene films. The metal films were deposited using the method of magnetron sputtering in an argon atmosphere. The film thickness was controlled by measurements on a Certus Standard V scanning atomic force microscope (NanoScanTechnology, Dolgoprudnyy, Russia), and was 100 ± 10 nm. The transfer was carried out on a HTF MARK nanosecond fiber laser (Bulat, Zelenograd, Russia) with a wavelength of 1064 nm, and a pulse duration of 100 ns. The energy in the pulse was 0.2 mJ. The radiation was focused on the film using an F-theta lens with a focal length of 160 mm. There was a gap of 1–2 mm between the metal film and the glass slide with bacterial biofilm on which the transfer was carried out (Figure 1) [10,11].

After treatment, the glass substrates with biofilms and nanoparticles deposited on them were placed in test tubes with distilled water. Then, the antibacterial properties of the nanoparticles were studied using seeding methods. The essence of the technique lies in the fact that the test sample is diluted in ten-fold steps, and sown on a nutrient-dense medium. Over time, the samples are examined, and it is established at what limiting dilution bacteria are detected. The Koch method is based on the inoculation of a certain volume of three final dilutions in three Petri dishes with a nutrient medium. In the final dilutions, there should not be too many cells for each of them to form an isolated colony, but not too few (if there are less than ten colonies in the Petri dish, then this result cannot be used for counting). Each isolated colony is the offspring of one cell, which makes it possible to correlate the number of grown colonies with the number of cells. A Drigalski spatula is usually used to spread the cells evenly. The colony-forming units (CFUs) are counted, which theoretically makes it possible to determine the concentration (number) of microorganisms per unit of volume. In our study, the determination of the viability of bacteria was carried out using the Live/Dead method. According to the manufacturer’s protocol for preparing a working solution of fluorescent dyes, we added 3 mL of SYTO^®^ 9 dye and 3 µL of propidium iodide dye to 1 mL of water (sterilized by a filter). Next, we applied the coloring solution very carefully so as not to disturb the biofilm on the biofilm sample. Then, we covered the staining dishes and incubated the sample for 20–30 min at room temperature, protecting it from light. We gently rinsed the sample with water, and then observed it through a microscope. The studies were carried out using a Nikon H600L microscope (Eclipse Ni, Tokyo, Japan) with a lens magnification of X 20. The excitation/emission maxima for these dyes are approximately 480/500 nm for the SYTO9 dye, and 490/635 nm for propidium iodide.

The topography of the samples was examined using a scanning electron microscope (SEM) TESCAN VEGA (Tescan, Brno, Czech Republic) with an R-STEM detector. For SEM characterization, nanoparticles were transferred to a polished silicon wafer, as well as to a silicon wafer with a daily bacterial biofilm grown on it.

The IR spectra of bacterial biofilms were studied before and after treatment with nanoparticles, by means of an FT-IR spectrophotometer V-70 (Bruker, Billerica, MA, USA) in the range of 800–4000 cm^−1^. The spectra of IR transmittance were converted to optical density, normalized to the optical density values of the substrate, and corrected for the baseline. The second derivatives were calculated in order to visualize the hidden peaks, with smoothing using the Savitzky–Golay method.

Raman analysis was performed in the range of 600–2000 cm^−1^, using a confocal Raman microscope Confotec MR 350 (Sol Instruments, Minsk, Belarus) at the excitation wavelength of 532 nm.

## 3. Results and Discussions

### 3.1. SEM Characterization

**Figure 2:**
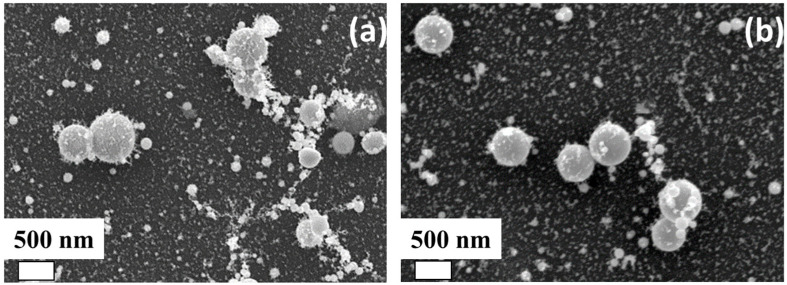
SEM visualization of (**a**) copper, and (**b**) silver NPs.

The nanoparticles resulting from laser transfer were rounded and not aggregated (Figure 2). The size of silver and copper nanoparticles varied from 20 to 600 nm. There was a polymer shell on the particles.

**Figure 3:**
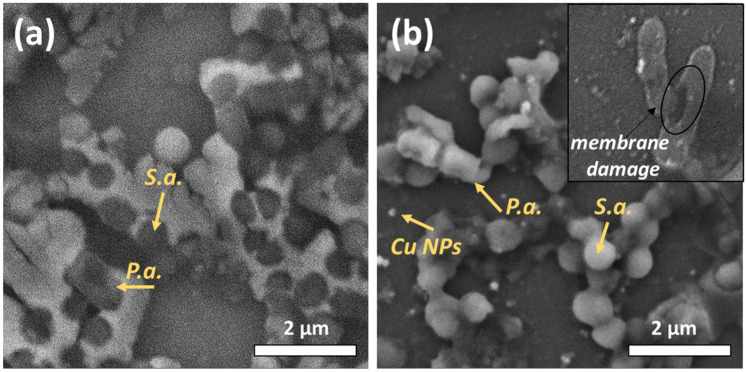
SEM visualization of mixed-species bacteria *Staphylococcus aureus*/*Pseudomonas aeruginosa* (**a**) before, and (**b**) after Cu NP treatment.

Figure 3 shows the SEM images of the mixed-species bacteria *Staphylococcus aureus*/*Pseudomonas aeruginosa* before and after their treatment with copper nanoparticles. The bacterium *Staphylococcus aureus* has a spherical shape, the size of which is in the range of 500–1000 nm. *Pseudomonas aeruginosa*, in turn, resembles elongated sticks (1–3 microns). After treatment with nanoparticles, a rupture of the bacterial membrane is observed.

**Figure 4:**
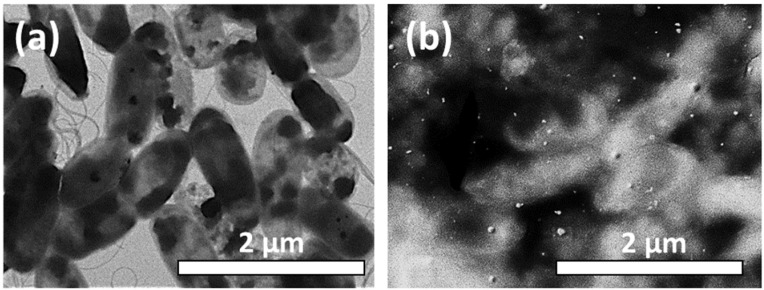
SEM visualization of *Pseudomonas aeruginosa* bacteria (**a**) before, and (**b**) after Cu NP treatment.

In addition, through the use of the R-STEM detector on a scanning electron microscope (Figure 4), the culture of *Pseudomonas aeruginosa* was visualized before and after its treatment with Cu NP nanoparticles. The images demonstrate that the nanoparticles covered the surface of the bacteria, and the internal structure of the bacteria changed.

### 3.2. Investigation of the Antibacterial Effect of Nanoparticles on Mixed-Species Biofilms

All samples with bacterial biofilms were grown in the same conditions. Three samples were processed for each type of biofilm. To determine the degree of the antibacterial effect of the nanoparticles on the biofilms, the experimental samples were compared with biofilms of untreated nanoparticles (control of biofilm growth). The averaging of results was performed over three repetitions. It can be seen that the number of bacteria decreased by several orders of magnitude. Silver and copper nanoparticles completely destroyed the biofilms *Staphylococcus aureus*, *Pseudomonas aeruginosa*, *E. coli*, and their mixed-species biofilms. The number of bacteria in these samples decreased by 5–8 orders of magnitude (Table 1).

**Figure 5:**
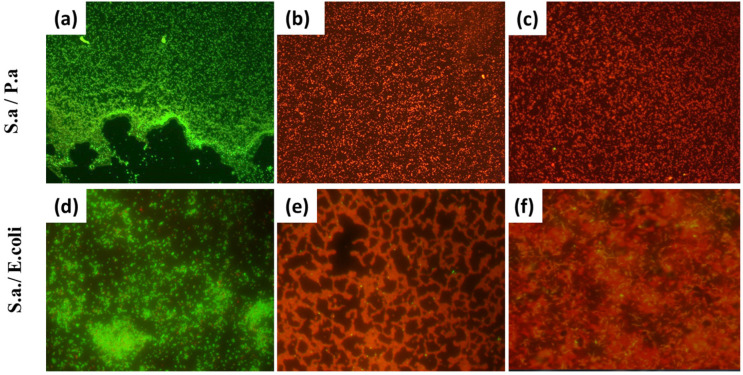
Results of Live/Dead for mixed-species bacteria biofilms: (**a**,**d**) control; (**b**,**e**) biofilms treated with Cu NPs; (**c**,**f**) treated with Ag NPs (the instrumental magnification is 600×; the image size is 30 × 25 µm).

We used the Live/Dead Viability/Cytotoxicity Kit, a fast and simple two-color assay to determine the viability of cells in a population based on plasma membrane integrity, to confirm the microbiological culture results. The method allows the distinguishing of living cells from dead cells via simultaneous staining with green fluorescent (Cyto-9) to indicate the activity of intracellular esterase, and red fluorescent (propidium iodide) as a marker of the loss of integrity of the plasma membrane. This method is highly sensitive, due to the bright fluorescence of both dyes when interacting with either live (for Cyto-9) or dead (for propidium iodide) cells. The bacterial biofilms were grown on glass slides. The control samples (Figure 5a,d), which were mixed (*Staphylococcus aureus* with *Pseudomonas aeruginosa* and *E. coli)* biofilms without nanoparticles applied on top, showed good bacterial survival (green color). Upon application of copper (Figure 5b,e) or silver (Figure 5c,f) nanoparticles, a stable red coloration of the samples was observed, which indicated the death of bacteria and a very significant bactericidal effect. These results are fully consistent with the results of the microbiological tests.

Preparations of silver and copper nanoparticles are widely used in medical practices as an alternative to traditional antimicrobial, antifungal, and disinfectant methods. Silver NPs and other nanomaterials are used in the creation of artificial bone implants, in dressings, in drug delivery, in designing biosensors, as contrast agents for visualization, etc. According to the literature, the toxicity of nanoparticles has not been detected within the oral and application methods of administering silver and copper nanoparticles. There is scientific evidence that nanoparticles are able to migrate to different organs, and overcome encephalitic and placental barriers, when administered intravenously. However, there are few data on the application method of administration. Moreover, a great number of registered dressings, ointments, and wound-washing products with silver nanoparticles are on sale. Essentially, the toxicity of nanoparticles is due to impurities. It is known that nanomaterials obtained using chemical methods may include impurities from the initial compounds used. Therefore, the physical method of obtaining low-frequency metals is considered the cleanest.

### 3.3. Raman and FTIR Spectroscopy of Mixed-Species Bacteria Staphylococcus aureus and Pseudomonas aeruginosa

Raman spectroscopy is an attractive method in the recognition of the causative agents of bacterial infections, and thereby of bacterial infections themselves [32].

**Figure 6:**
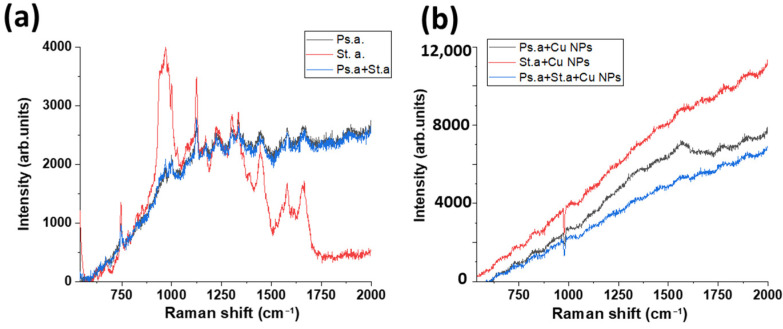
Raman spectra of *Staphylococcus aureus* and *Pseudomonas aeruginosa* bacteria (**a**) before and (**b**) after Cu NP treatment.

In order to take the Raman spectra, the samples were placed directly under a 50× microscope objective, and the laser spot was focused sequentially on the central surface of the bacterial colony. It was decided that the spectral range would be from 700 to 2000 cm^−1^ to cover the most suitable zone. Figure 6 demonstrates the Raman spectra of *Staphylococcus aureus*, *Pseudomonas aeruginosa*, and their mixture before and after treatment with copper nanoparticles. Prior to treatment with nanoparticles, the main peaks characteristic of either one or the other type of bacteria were clearly visible in the spectra (presented in Table 2). After processing, a strong luminescence occured.

**Figure 7:**
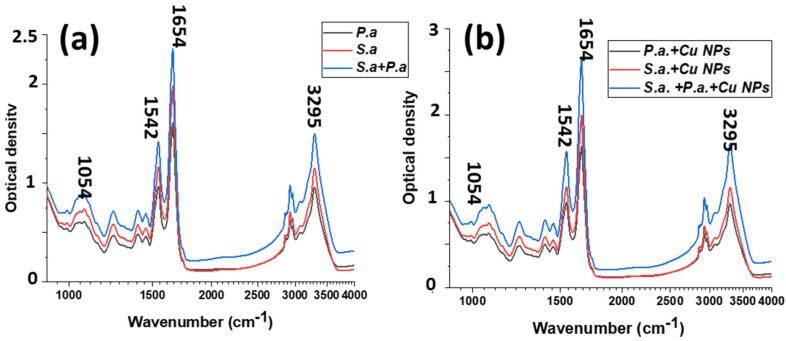
FTIR spectra of *Staphylococcus aureus* and *Pseudomonas aeruginosa* bacteria (**a**) before and (**b**) after Cu NP treatment.

FTIR spectroscopy is a unique and non-destructive method for molecular composition and dynamics analysis [33]. In our work, FTIR spectra were taken for the bacteria *Pseudomonas aeruginosa, Staphylococcus aureus*, and their mixtures. Spectra were also taken for these bacteria when treated with copper nanoparticles (Figure 7).

Table 3 lists the main peaks observed in the spectra, and their descriptions. The spectral region of about 3000 cm^−1^ is associated with the symmetric and asymmetric stretching vibrations of -CH_3_ and >CH_2_. The 1500–1800 cm^−1^ region contains the amide I and amide II bands. These peaks provide information about the protein structure.

A slight shift in frequencies was detected for the signals associated with the OH functional group, a 997 cm^−1^ (COH bending) and a 1157 cm^−1^ (C-O stretching), which evidently indicate the interaction of OH groups with the Cu NPs [34].

**Figure 8:**
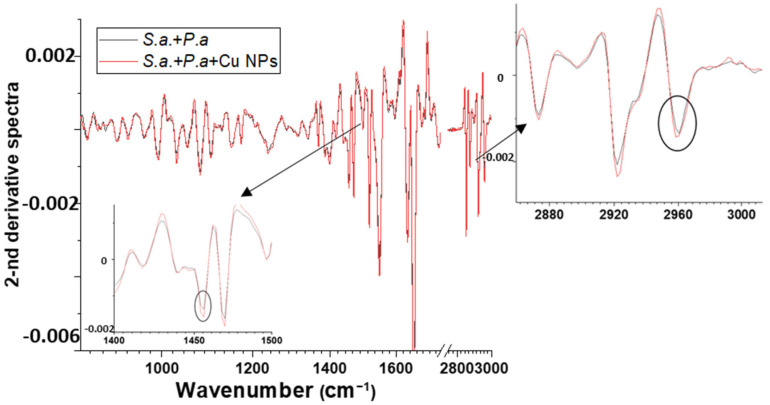
Second-derivative FTIR spectra of mixed-species bacteria *Pseudomonas aeruginosa, Staphylococcus aureus*.

For mixed biofilms, the second derivative was considered for more detailed analysis and the detection of hidden peaks (Figure 8).

The broadening peak ≈1450 cm^–1^ (C-H (of CH_2_)) corresponding to molecular vibrations in lipids may indicate the disruption of the lipid structure. After the Cu NP treatment, there was a slight shift in the FTIR spectrum, in the region ≈2960 cm^−1^, which corresponds to fatty acids that present in the membrane. This shift could indicate the decrease in the relative motional freedom of molecules [35].

## 4. Conclusions

In this work, we investigated the effect of copper and silver nanoparticles obtained by the LIFT method on naturally occurring mixed-species biofilms of gram-positive and gram-negative bacteria. According to the results of microbiological seeding, and the results of the Live/Dead method, a complete suppression of mixed-species bacterial biofilms was shown. Raman studies showed strong luminescence for bacteria treated with copper nanoparticles. The mechanism of action of nanoparticles mainly consists of direct contact with the bacterial cell wall, and the formation of reactive oxygen species. The FTIR spectra showed some shifts in individual peaks, which are indicative of the disruption of the lipid structure. This method could show promise for the processing of food surfaces in production, as well as for the treatment of wound infections. In the near future, we plan to conduct experiments around the application transfer of nanoparticles to infected wounds. The experiments will be conducted on laboratory mice. Not only will the wound-healing effect and the general condition of the animals be evaluated, but also the migration of nanoparticles to different organs.

## Figures and Tables

**Table 1:** Effect of transferred NPs on CFU/mL values of clinical isolates biofilms.

Bacteria	Ag NPs	Cu NPs	Control
***S. aureus***	<1	<1	4 × 10^6^
***P. aeruginosa***	<1	<1	3 × 10^7^
***P. aeruginosa + E. coli***	<5	<5	8 × 10^7^
***S. aureus + E. coli***	<5	<5	2 × 10^7^
***S. aureus + P. aeruginosa***	<5	<5	1 × 10^5^

**Table 2:** Raman spectra assignments.

Peak (cm^−1^)	Assignments
745	Ring breathing mode of DNA/ RNA bases thymine
1120	C–C and C–N assigned to adenine/thymine and stretching mode of amide III
1158	C-C, C-N stretching (protein)
1307	CH_2_ deformation in proteins and lipids
1447	CH_2_ deformation, CH_3_/CH_2_ twisting in carbohydrates, proteins and lipids
1578	Ring breathing modes in the DNA bases
1653	Stretching mode of amide I

**Table 3:** FTIR spectra assignments.

Peak (cm^−1^)	Assignments
1054	P=O stretches of phosphodiesters
1236	P=O stretches of phosphodiesters
1454	CH_3_ asymmetric and symmetric deformation of proteins
1545	Amide II bands, referred to N-H deformation of amides associated with proteins
1654	Amide I band, C=O stretching vibrations of amides associated with proteins
1800–1400	Vibrational feature of group protein-associated
2960	C-H str (asym) in CH_3_ of fatty acids
3290	O-H stretch

## Data Availability

The data presented in this study are available on request from the corresponding author.
